# Supplementary figures and images for: NK and T Cell Immunological Signatures in Hospitalized Patients with COVID-19
Source: Cells. 2021 Nov 15;10(11):3182. doi: 10.3390/cells10113182 (PMC8618013; doi:10.3390/cells10113182)

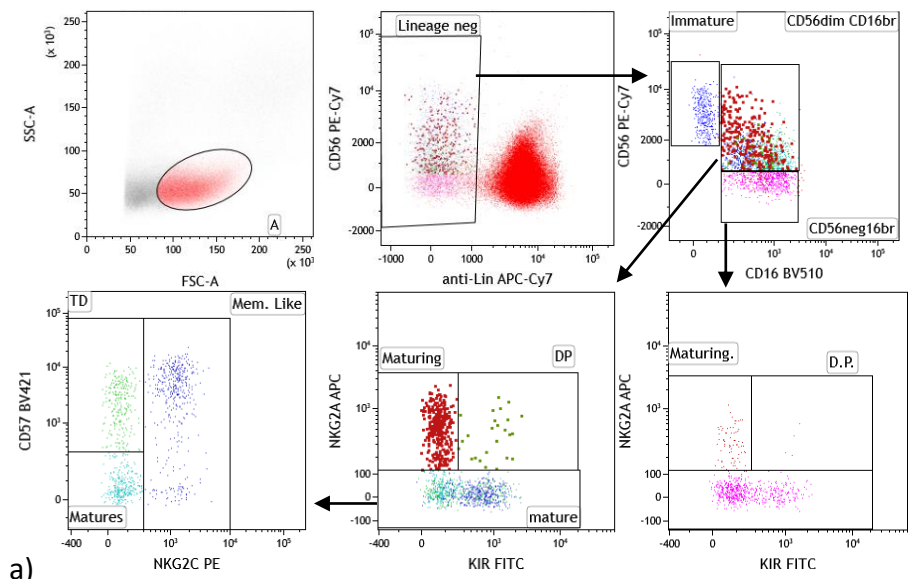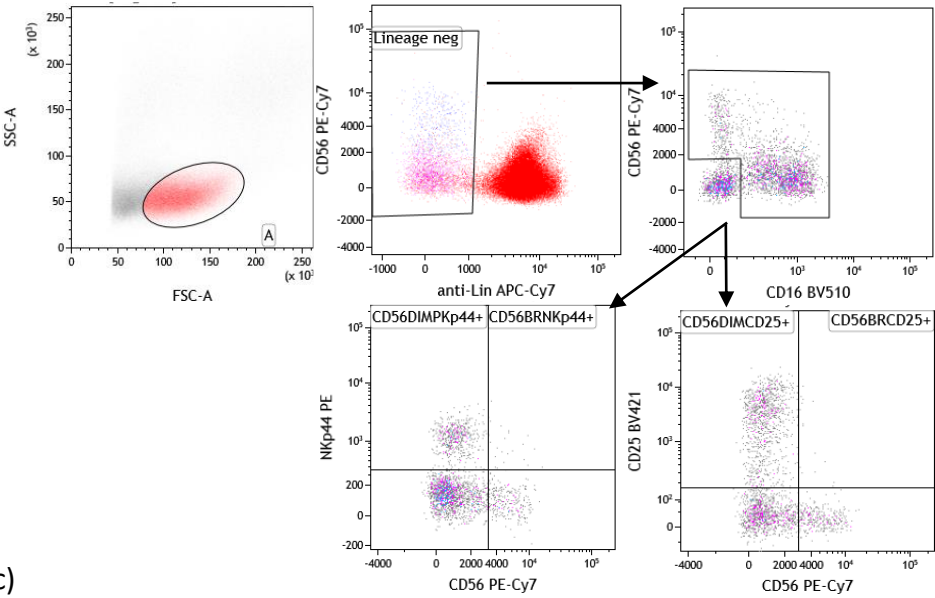

Supl.Fig.1 Gate strategy of NK cells tube 1 (a), 2 (b) and 3 (c) and T maturation.

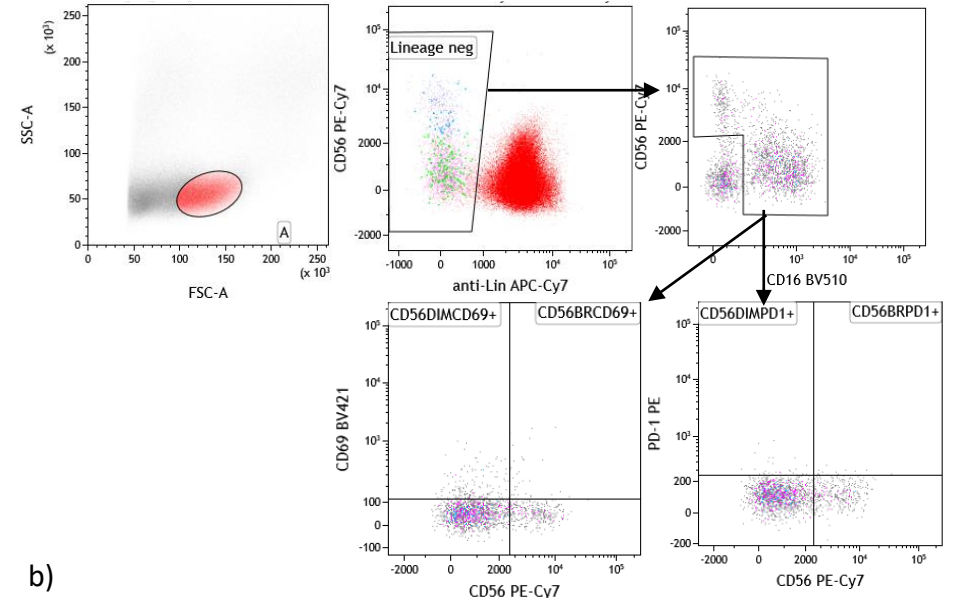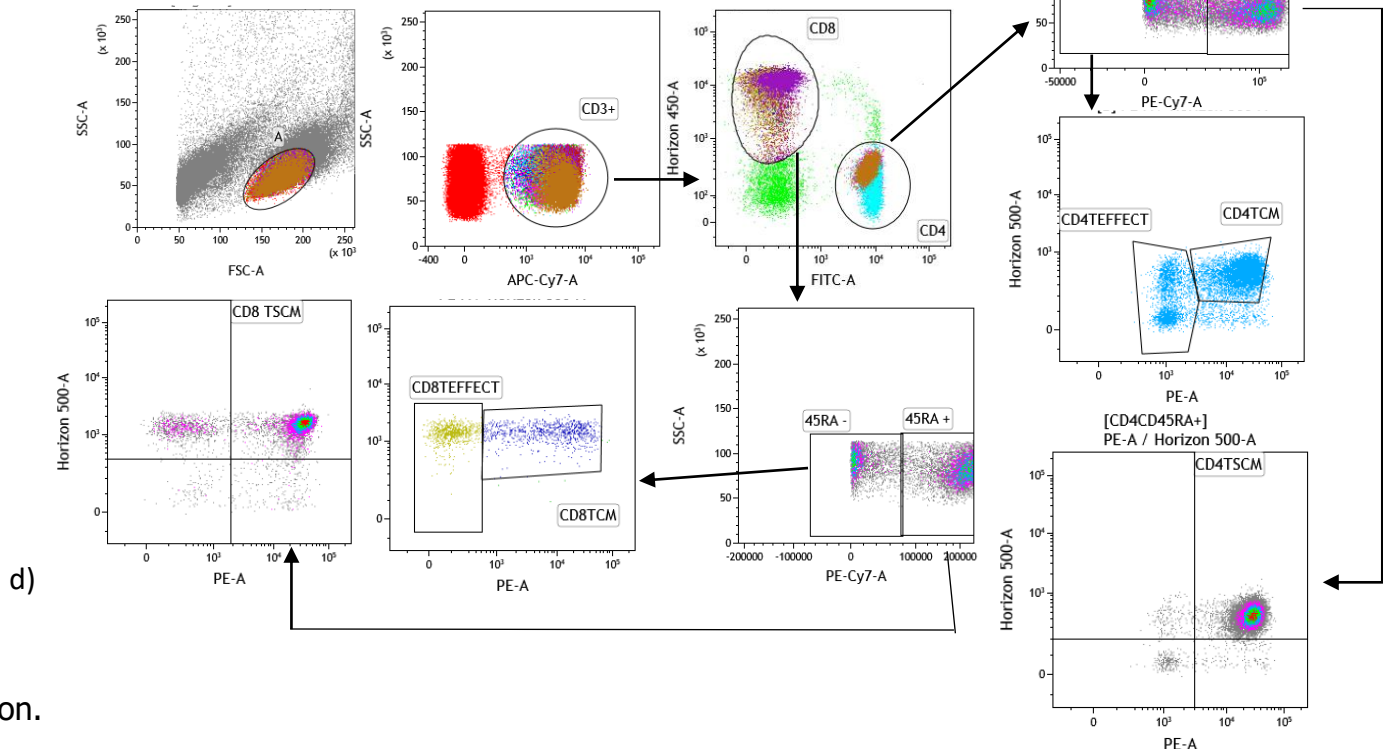

Supplement: Supplementary file 1 [file cells-10-03182-s001.zip › cells-1448787-supplementary.pdf]
